# Error Propagation Analysis for Quantitative Intracellular Metabolomics

**DOI:** 10.3390/metabo2041012

**Published:** 2012-11-21

**Authors:** Jana Tillack, Nicole Paczia, Katharina Nöh, Wolfgang Wiechert, Stephan Noack

**Affiliations:** 1 Institute of Bio-and Geosciences, IBG-1: Biotechnology, Forschungszentrum Jülich GmbH, 52425 Jülich, Germany; Email: j.tillack@fz-juelich.de (J.T.); n.paczia@fz-juelich.de (N.P.); k.noeh@fz-juelich.de (K.N.); w.wiechert@fz-juelich.de (W.W.); 2 JARA-High Performance Computing, Forschungszentrum Jülich GmbH, 52425 Jülich, Germany

**Keywords:** metabolomics, quantification, intracellular metabolites, quenching, metabolite leakage, LC-MS/MS, error propagation analysis, *Corynebacterium glutamicum*

## Abstract

Model-based analyses have become an integral part of modern metabolic engineering and systems biology in order to gain knowledge about complex and not directly observable cellular processes. For quantitative analyses, not only experimental data, but also measurement errors, play a crucial role. The total measurement error of any analytical protocol is the result of an accumulation of single errors introduced by several processing steps. Here, we present a framework for the quantification of intracellular metabolites, including error propagation during metabolome sample processing. Focusing on one specific protocol, we comprehensively investigate all currently known and accessible factors that ultimately impact the accuracy of intracellular metabolite concentration data. All intermediate steps are modeled, and their uncertainty with respect to the final concentration data is rigorously quantified. Finally, on the basis of a comprehensive metabolome dataset of *Corynebacterium glutamicum*, an integrated error propagation analysis for all parts of the model is conducted, and the most critical steps for intracellular metabolite quantification are detected.

## Abbreviation

**Table:** 

Input variable	Symbol
Cell dry weight	*c_CDW_*
Cell dry weight specific biovolume	*v_CDW_*
Volume-specific biovolume	*v_brs_*
Bioreactor sample volume	*V_brs_*
Cytosolic volume	*V_cyt_*
Volume of the extraction reagent	*V_exc_*
Total extraction volume	*V_exc_*
Residual quenching volume after cell separation	*V_res_*
Volume of the quenching reagent	*V_que_*
Metabolite concentration of the standard	*c_std_*
Metabolite concentration in the extract	*c_exc_*
Cytosolic metabolite concentration	*c_cyt_*
Metabolite concentration in the quenching supernatant	*c_qsn_*
Metabolite concentration in the culture broth	*c_cub_*
Leakage concentration	*c_lea_*
Peak area quotient of respective sample type	*𝜐_[…]_*
^12^C peak area of respective sample type	*η_12C,[…]_*
^13^C peak area of respective sample type	*η_13C,[…]_*

## 1. Introduction

Quantitative “omics” technologies, such as metabolomics, play a key role in driving systems biology towards becoming an applied science for metabolic engineering and synthetic biology of microorganisms. Providing quantitative data of the cell’s transcriptome, proteome, metabolome and fluxome provides the opportunity to unravel piecewise the complex regulatory mechanisms underlying all *in vivo* metabolic processes. Moreover, it is intuitively obvious that the integration of such multi-omics data into a mathematical model is a prerequisite for extracting hidden information on regulatory effects constituting the cellular metabolism [1]. This is especially true of all approaches aiming for mechanistic descriptions of metabolic processes, such as transcription, translation and enzyme catalysis [2]. 

**Figure 1:**
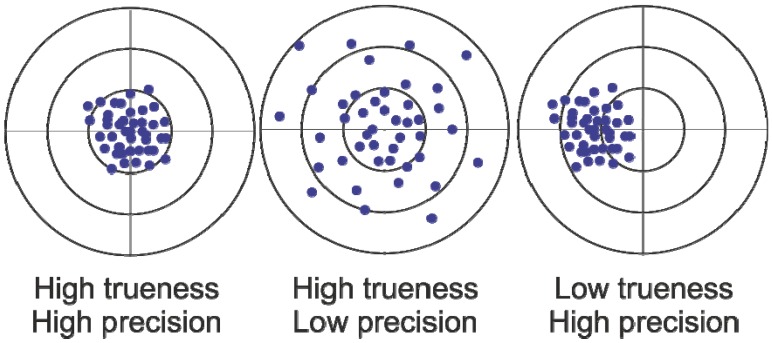
Schematic view of the terms “trueness” and “precision” of a measurement.

However, a meaningful interpretation of multi-omics data depends to a great extent on the quality of the data itself. Here, it should be remembered that the term “accuracy” refers to both the “trueness” and “precision” of a measurement (Figure 1). While “trueness” defines the systematic deviation of an obtained measurement value from the required “true” value, the term “precision” is linked to the reproducibility of an analytical protocol, *i.e.*, the sum of randomly distributed errors due to handling steps around the actual measurement. Both terms are equivalent to “bias” and “variance” in statistics.

Clearly, from a scientific point of view, “trueness” is most important, because measurements in scientific experiments should create an exact image of reality in order to be useful for generating and testing hypotheses. In the field of biotechnology, and especially when dealing with the analysis of complex biological samples of specific cultivation experiments, this is a rather difficult task. Here, many factors exist that are related to the technical (e.g., bioreactor device) and biological (e.g., cellular growth) part of an experiment, and only some of them are accessible.

This contribution concentrates on metabolomics, which generally attempts to study metabolites and their concentrations, interactions and dynamics within complex samples [3]. The variability of chemical properties, as well as diverse concentration ranges (from picomole to millimole) within the metabolome, poses great challenges for intracellular metabolite quantification, and various protocols for sample preparation are available [4].

In order to obtain accurate intracellular concentration data, repetitive experiments, including all sample processing steps (biological replicates), are necessary. Unfortunately, this is either too laborious and time-consuming or simply not possible for biological reasons. Hence, these protocols are usually only repeated in selected parts (applying “technical replicates”) to obtain at least some rough estimate of data accuracy. 

Thus, our major objective is to derive an analysis tool for handling error propagation during metabolome sample processing. This is done by formulating a process model for the complete workflow for the quantification of intracellular metabolites in biomass-containing samples from cultivation experiments. By focusing on one specific protocol, we comprehensively investigated all currently known and accessible factors that ultimately determine the accuracy of intracellular metabolite concentration data. We demonstrate that the resulting model allows a trustworthy estimation of intracellular concentration data and is a viable basis for the further improvement of any sample processing protocol.

## 2. Results and Discussion

### 2.1. Sample Processing for Quantitative Intracellular Metabolomics

**Figure 2:**
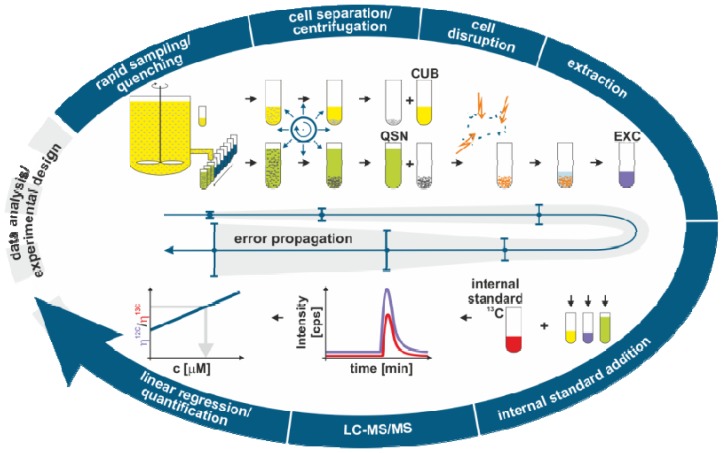
Scheme of different sample processing steps for intracellular metabolome analysis. In each step, a number of influencing variables are involved that are all subject to systematic and random-based errors. Hence, in order to maximize the overall accuracy of intracellular metabolite data, variables exerting the greatest influence have to be identified and the respective errors minimized. Abbreviations: QSN, quenching supernatant sample; CUB, culture broth sample; EXC, extract sample.

Figure 2 gives an overview of our standard sample processing protocol for intracellular metabolome analysis. In the following, we will briefly describe all individual steps, focusing on the most relevant error sources. This description then provides the theoretical background for the formulation of a process model covering the whole procedure, ranging from biomass determination to mass spectrometry analytics.

#### 2.1.1. Biomass Determination

Biomass determination is usually not considered to be a step in sample preparation for metabolome analysis. However, it significantly affects the final results by defining the:

- appropriate volume for the metabolome sample,
- reference value for the resulting intracellular metabolite concentrations.

In particular, it has to be kept in mind that the only relevant biomass for intracellular metabolite quantification is given by the sum of all cells with intact membranes containing the “biovolume” [5]. However, in many studies it is not the actual measured biovolume, but rather a rough estimate based on the measured cell dry weight and a literature value for the specific cell volume that serves as the reference value for the absolute amount of intracellular metabolites [6].

#### 2.1.2. Metabolome Sampling

The main requirements for a sampling technique suitable for metabolome analysis are:

- exact withdrawal of the pre-defined sample volume,
- no time delay between sampling and inactivation of metabolism (quenching),
- no sample contamination.

Apart from manual sampling using syringes [7], a large number of different automatic sampling devices have been developed in the past few decades [8]. These are either based on active pumping systems or on valve controls driven by overpressure or underpressure.

The resulting sample volume is inherently subject to a systematic deviation from the set-point value, depending on the specific sampling device. In the case of valve controls, this deviation is mainly a function of the valve opening time and the actual pressure inside the bioreactor, which can be quite high. In contrast, pumping systems allow the withdrawal of precise sample volumes. However, depending on the pipe lengths, the time delay between sampling and quenching, as well as the dead volume leading to sample contamination, is comparatively high. 

#### 2.1.3. Quenching

The ultimate challenge in the quantification of intracellular metabolites is their low chemical stability, as well as fast enzymatic conversion by the metabolic reactions under investigation. In recent years, a variety of different quenching protocols has been proposed [9], and quenching with the help of cold methanol solutions has become the standard procedure for bacteria. In this case, the sample is directly transferred into a defined volume of quenching solution consisting of 50–100% methanol with varying supplements, which is cooled down in the range of −20 °C to −80 °C [10,11,12].

Nowadays, it is apparent that microorganisms can lose significant amounts of intracellular metabolites during quenching, but it is still an open question whether the shift of temperature, the osmotic pressure, the influence of the organic solvent or a certain combination of all factors leads to the underlying change in cell membrane integrity [13]. Clearly, the leakage effect can falsify the image of the metabolic state of a cell dramatically. 

Hence, an appropriate quenching procedure should allow

- fast inactivation of the cell’s metabolism in a state which is as close as possible to the *in vivo* state during cultivation,
- correction of metabolite loss as a result of leakage.

In order to quantify and, if necessary, correct for metabolite leakage, the resulting quenching supernatant after the subsequent cell separation step is analyzed (cf. Figure 2, QSN sample in addition to the EXC sample). In addition, a cell-free sample of the culture broth is taken (cf. Figure 2, CUB sample) to account for the amounts of metabolites already present in the culture media during sampling [14]. By taking this extended version of the classical quenching approach, three error-prone concentration measurements for each metabolite are necessary to calculate the final intracellular concentration. 

#### 2.1.4. Cell Separation

The separation of the quenched cells from the extracellular medium depends on the criteria of

- fast separation to minimize the dwell time of cells in the quenching solution,
- complete separation with minimal physical energy input.

In general, cell separation is carried out via centrifugation or filtration. In both cases, the minimal separation time is a compromise to fulfill both criteria [7], and therefore, a significant effect of leakage per se cannot be prevented.

Irrespective of the method applied, a certain amount of extracellular volume is present between the cells (intercellularly), attached to vessel walls or in the membrane pores (dead volume). Therefore, the sample always becomes contaminated, either by increasing or diluting the resulting amount of intracellular metabolites obtained after extraction.

#### 2.1.5. Cell Disruption and Extraction

In the next step, the intracellular metabolites have to be extracted from the cytosol. For this task, an integrated method combining cell disruption and immediate metabolite extraction is usually applied that should primarily ensure

- complete cell disruption,
- extraction of the complete amount of all metabolites of interest,
- no degradation or chemical modification of metabolites,
- compatibility of all solvents with subsequent analytical techniques.

On the one hand, depending on the organism and its cell wall characteristics, different methods for cell disruption have been developed covering physical (temperature, ultrasonic, milling, etc*.*), chemical (chloroform, etc*.*) or biological (enzymatic digestion) treatments [15]. However, in most cases, and depending on the degree of cell disruption, the mixture of the whole cytosol (biovolume) with the extraction reagent is incomplete. 

On the other hand, a great number of extraction reagents have proven suitable for prokaryotes [11,12,16]. However, all methods suffer from their more or less undefined specificity and, hence, extraction efficiency for certain metabolites [15].

#### 2.1.6. Analysis

In the last step of the sample processing protocol, the intracellular metabolites are quantified by applying a measurement method that provides

- high sensitivity and selectivity,
- wide linear dynamic range and broad analytical spectrum,
- quantification via available standards.

Due to its comparatively good performance with respect to all the above-mentioned criteria, the LC-ESI-MS/MS technique has been favored for quantitative metabolomics in recent years [17]. Nevertheless, it is also apparent that, depending on the complexity (coeluents) and chemical properties (pH, polarity and salt concentration) of the biological matrix, ion suppression can be a serious problem, especially for ESI-MS based methods [18]. Consequently, the resulting peak intensity or area is not directly proportional to the amount of a certain metabolite to be quantified.

### 2.2. Modeling the Metabolome Sample Processing

The experimental protocol described in the previous sections finally results in peak area measurements of respective metabolites in the different process samples. In the following, a reference model for the metabolome sample processing is introduced, and the steps required converting the measured peak areas into intracellular metabolite concentrations are described, proceeding in reverse order through the experimental workflow. This reference model will later be refined by correcting for systematic errors that are known and accessible during sample processing.

#### 2.2.1. Estimation of Extract Concentrations

In the first step, the measured peak area for a specific metabolite is transformed into the corresponding concentration value related to the total extraction volume of the extract sample. Following the classical external calibration approach, samples with defined metabolite concentrations *c_i,std_* are measured, and a linear model for the resulting standard peak areas *η_i,std_* is formulated:

*η_i,std_* = *c_i,std_* · *m* + *n* (1)

The model parameters comprising slope *m* and intercept *n* are estimated by, for example, applying weighted linear least-squares regression utilizing the variances of the standard measurements as weights [19]. 

By transforming the model in Equation (1), the extract concentration *c_exc_* can be estimated from the measured peak area *η_exc_* of the metabolite in the extract sample:

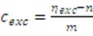
(2)


#### 2.2.2. Estimation of Cytosolic Concentrations

In order to obtain intracellular concentration data related to the cytosolic volume of extracted cells, the estimated extract concentration from Equation (2) has to be further converted by taking into account the dilution during biomass extraction. 

Usually, the cytosolic volume *V_cyt_* is estimated from the measured cell dry weight *c_CDW_* in the bioreactor sample of a volume *V_brs_* defined *a priori* and the specific biovolume *v_CDW_* taken from the literature:

*V_cyt_* = *c_CDW_* · *V_brs_* · *v_CDW_* (3)

In that case, *V_cyt_* does not reflect the actual biovolume, because the value of *v_CDW_* is not constant, but is highly dependent on the state of cultivation during metabolome sampling.

The total extraction volume 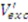 is then estimated from the cytosolic volume *V_cyt_* of disrupted cells and the volume of the extraction reagent *V_exc_* defined *a priori:*

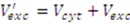
(4)


Finally, the cytosolic concentration *c_cyt_* can be estimated as:

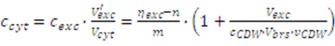
(5)


At this point, we obtained an intracellular concentration value that is most likely far from the true *in vivo* value, due to some uncertainties (e.g., matrix effects, metabolite leakage, *etc.*) that are not covered by the reference model introduced so far.

### 2.3. Correction for Systematic Errors

In general, the experimental workflow as described above involves process quantities and related systematic errors (biases) that are:

- independent of the actual experiment, e.g., the pre-defined sample volume *V_brs_* (cf. Equation (3)): in this case, the bias from a certain set-point value can be determined in a separate experiment (Table 1),
- dependent on the actual experiment, e.g., the final intracellular concentration *c_cyt_* (cf. Equation (5)): hence, for bias correction, additional measurements or internal standards are needed.

With respect to the second case, the reference model described above will be refined in the following by correcting for all accessible biases that are inherent in the sample processing protocol. 

#### 2.3.1. Matrix Effects

Currently, the method of choice to correct for analytical biases as a result of matrix effects (e.g., ion suppression) is isotope dilution mass spectrometry (IDMS) [20]. In short, a prepared mixture containing the uniformly ^13^C-labeled analogs of all target metabolites is spiked into each metabolome sample (including standards) before LC-MS/MS measurement and is analyzed in parallel. This allows the estimation of peak area quotients *𝜐* of the respective ^12^C and ^13^C analytes:

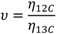
(6)


Assuming that both analytes, *i.e.*, ^12^C and ^13^C, show identical behavior regarding the underlying matrix effects, the quotient *𝜐* of the resulting peak areas automatically corrects for this kind of bias and Equation (1) and (2) can be reformulated as:

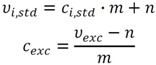
(7)


It should be noted that IDMS only provides trustworthy results if ^12^C analyte and its ^13^C analogon are ionized in an identical manner and, hence, are subject to the same ion suppression. This assumption is only valid in the linear measurement range where the ion source is not saturated with any kind of ionizable substance [21].

#### 2.3.2. Measurement of Biovolume

Since the actual biovolume of the biomass in the quenching sample is the relevant reference volume for the amount of intracellular metabolites, a direct and experiment-specific measurement is highly desirable. An appropriate Coulter counter method, for example, can be used to measure the biovolume *v_brs_*, and the cytosolic volume is more accurately estimated by:

*V_cyt_* = *V_brs_* · *v_brs_* (8)

#### 2.3.3. Incomplete Cell Separation

Due to incomplete cell separation (subsequent to centrifugation or filtration), there is always some quenching supernatant left in the biomass sample prior to the extraction step. First, this leads to an increase in the total extraction volume already introduced in Equation (4):

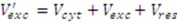
(9)


Here, the term *V_res_* denotes the residual quenching volume after cell separation, causing an additional dilution of the resulting extract concentration.

Second, the metabolites present in the quenching supernatant lead to a false-to-high estimation of the corresponding extract concentration *c_exc_* when applying Equation (7). In order to correct for this bias, the metabolite concentration in the quenching supernatant *c_qsn_* is estimated according to Equation (7). Considering the dilution by the extract volume leads to:

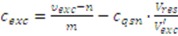
(10)


It should be noted that the obtained extract concentration might still be significantly biased due to the metabolite-specific extraction efficiency, which depends on the degree of cell disruption and the properties of the extraction reagent. In principle, this bias could be corrected by using an appropriate internal standard. However, no such standard is currently available, and therefore, the impact of incomplete extraction is not included in our model.

#### 2.3.4. Metabolite Leakage

As a final correction step, the potential metabolite loss due to leakage is considered. The proposed procedure relies on the additional determination of the culture broth concentration, *c_cub_*, which is estimated according to Equation (7). Together with the estimates already introduced for the extract and quenching supernatant concentrations (cf. Equation 10), and accounting for the respective sample dilutions, the leakage concentration can be estimated as:

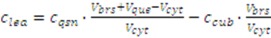
(11)


It should be noted that by formulating the mass balance of Equation (11), it is assumed that leakage is a result of metabolite loss via the cell membrane, e.g., by enforced diffusion, and not a consequence of cell damage, including loss of cytosolic volume.

Finally, modifying Equation (5) by taking the leakage concentration into consideration yields a more accurate estimate for the cytosolic metabolite concentration:

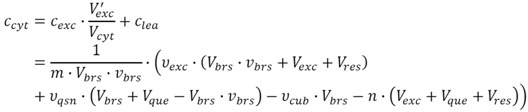
(12)


### 2.4. Application Example

The final model for metabolome data processing includes the basic equations for raw-data conversion, as well as the extensions for the correction of systematic errors (cf. Equation 12). In the following example, the process model is applied to a dataset from a cultivation experiment with a *Corynebacterium glutamicum* strain (see Experimental Section for more details). The dataset contained 44 measurements for metabolites from the central metabolism in three different samples (extract, culture broth and quenching supernatant) for one state of cultivation (mid-exponential phase).

#### 2.4.1. Linearity Check

**Figure 3:**
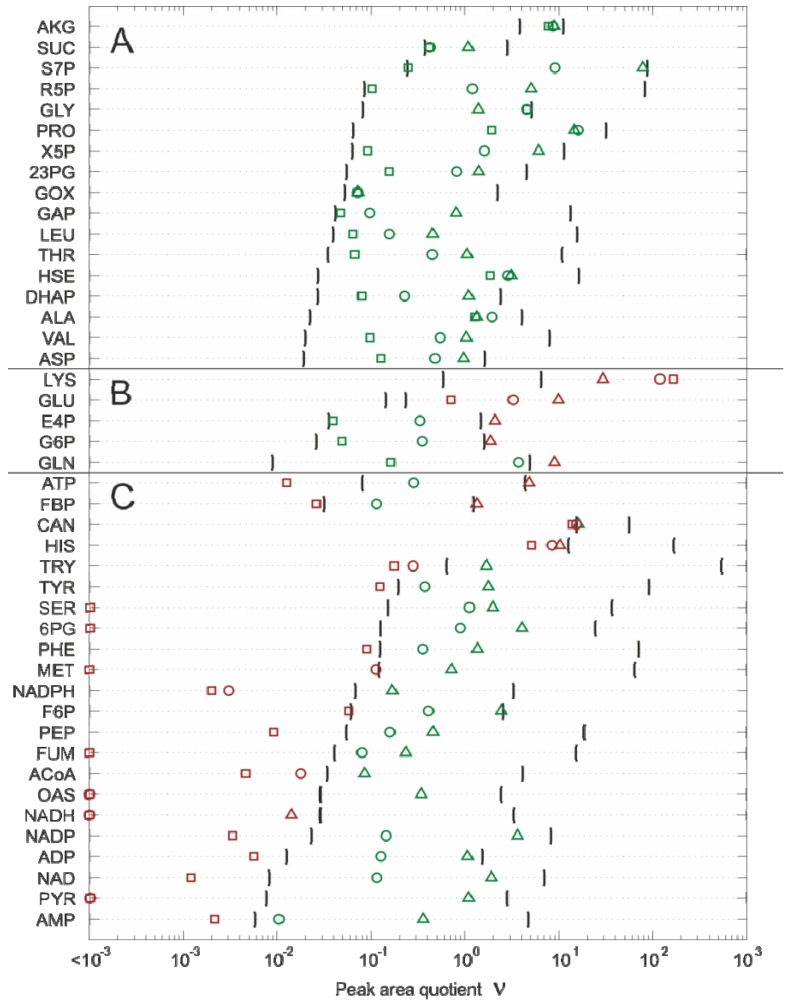
Linearity check for the resulting peak area quotients of metabolites in the three process samples of the dataset. The vertical black lines symbolize the lower and upper limits of the linear measurement range. The three symbols represent the peak area quotients for the extract (triangle), the quenching supernatant (circle) and the culture broth (square) sample. Green and red symbols indicate that the underlying measurement is inside or outside the linear range, respectively. Accordingly, all metabolites are arranged in three sections: (**A**) The dataset can be directly used for further processing; (**B**) Some samples need further dilution and re-measurement; (**C**) At least one peak area quotient is below the lower bound, and hence, the metabolite cannot be quantified with the applied protocol.

As a preliminary step, the data was filtered according to the linear measurement ranges resulting from the external calibrations with the appropriate standard quantifiers (Figure 3). The linearity check for this dataset leads to the following results:

- 17 metabolites can be further processed without any restriction.
- For five metabolites, the upper linear measurement range is violated and further sample dilution is necessary.
- In the case of 22 metabolites, the lower linear measurement range is violated, indicating that the respective datasets cannot be further processed.

On the basis of the 17 metabolites validated for further processing, the effect of each single bias correction step as discussed above was investigated.

#### 2.4.2. Effect of Bias Correction

**Figure 4:**
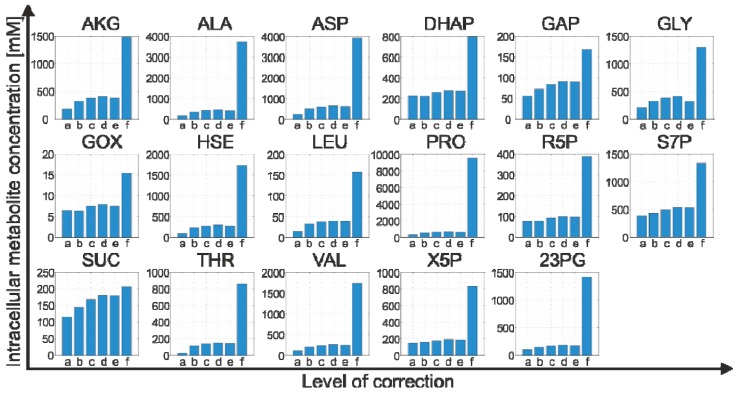
Effect of each bias correction step on the intracellular metabolite concentration. (**a**) reference model; (**b**) matrix effects; (**c**) measurement of biovolume; (**d**) incomplete cell separation causing metabolite dilution; (**e**) incomplete cell separation causing metabolite dilution and carryover; (**f**) metabolite leakage.

Starting with the reference model without any bias correction (cf. Equation 5), the change in the estimated absolute value for each metabolite concentration was simulated by successively considering all correction steps (cf. Eqations 6–12). The simulation results are shown in Figure 4, and it can be seen that:

- For 15 of the 17 metabolites, the use of the internal standard (IDMS) leads to an increase of the intracellular metabolite concentration.
- As expected, the actual measurement of the specific biovolume (*v_brs_*) leads to a smaller total cytosolic volume and, hence, results in an increase of the intracellular metabolite concentration.
- Interestingly, for nearly all metabolites, the consideration of the residual quenching volume after cell separation (*V_res_*) leads to only small changes in the intracellular concentration values. This can be easily explained by the opposing effect of metabolite dilution and carryover, as discussed previously in connection with Equation 9 and 10.

Finally, it can be clearly demonstrated that of all the processing steps, leakage correction leads to the most significant change in the intracellular concentration value. More specifically, without leakage correction, the intracellular metabolite concentrations are always estimated false-to-low.

The refined process model allows the estimation of intracellular metabolite concentrations reflecting the true *in vivo* state of the cell’s metabolome in the best way currently possible when applying our specific protocol.

#### 2.4.3. Propagation of Random Errors

This model is then applied to estimate the precision of the resulting concentration data by integrating the random errors of all process quantities (input variables) following an error propagation analysis. Due to the non-linearity of the model, a non-linear error propagation method should also be preferred. Therefore, we decided to use a classical bootstrap method [22]. Briefly, bootstrapping is a re-sampling method and can be understood as generating multiple biological replicates *in silico*, instead of performing the same experiments in the lab. By assuming that the random errors of all input variables are normally distributed with known mean and variance, the process model is applied several times for a set of input variables that is randomly added with noise. The propagation of these errors through the model then results in a probability distribution for the output variable (intracellular concentration), allowing us to derive their mean and variance.

Corresponding variances for all input variables were estimated in well-defined experiments (see Experimental Section for more details) and shown in Table 1. The resulting errors are in the range of 0.78% (quenching volume *V_que_*) to 19.05% (residual quenching volume after cell separation *V_res_*).

**Table 1:** Input variables of the metabolome sample processing protocol. Systematic (bias) and random (variance) errors were determined independently for each variable.

Input variable	Set point	Measurement value	Bias	Variance	Std. deviation [%]
*c_CDW_* [g L^−1^]	-	3.95	-	0.03	4.38
*v_CDW_* [µL mg^−1^]	-	1.93^a^	-	0.93	49.97
*v_brs_* [µL mL^−1^]	-	5.205	n.d.^b^	0.01	1.92
*V_brs_* [µL]	5000	4782	218	1463.83	0.80
*V_exc_* [µL]	1350	1372.275	22.275	303.74	1.27
*V_res_* [µL]	-	100.61	n.d.	367.49	19.05
*V_que_* [µL]	15000	15472.5	472.5	14565.11	0.78
*η_12C,std_0.25_* [counts]	-	4.29E+06	n.d.	2.79E+10	3.89
*η_12C,std_1_* [counts]	-	6.94E+06	n.d.	1.85E+11	6.20
*η_12C,std_5_* [counts]	-	1.64E+07	n.d.	5.26E+11	4.42
*η_13C,std_0.25_* [counts]	-	6.63E+06	n.d.	4.20E+09	0.98
*η_13C,std_1_* [counts]	-	6.24E+06	n.d.	1.66E+10	2.06
*η_13C,std_5_* [counts]	-	6.18E+06	n.d.	9.88E+09	1.61

^a^taken from [23], ^b^not determinable

In Table 2, the estimated intracellular metabolite concentrations from the reference model are compared to the concentration data obtained when applying the refined model, including bias correction and error propagation analysis. It can be easily seen that both datasets are significantly different, thus emphasizing the necessity of accurate data processing during metabolome analysis.

**Table 2:** Estimated intracellular metabolite concentrations when applying two different approaches of metabolome data processing.

Metabolite	Intracellular metabolite concentration [µM]	
	Reference model (Equation 5)	Refined model (Equation 12)
GAP	55.59	168.21 ± 8.67
DHAP	225.09	795.63 ± 40.97
23PG	96.16	1415.56 ± 72.80
R5P	77.10	388.75 ± 16.48
X5P	143.75	827.88 ± 36.65
S7P	386.17	1340.28 ± 53.80
AKG	188.87	1485.31 ± 451.76
SUC	115.68	205.45 ± 69.68
GOX	6.48	15.38 ± 13.82
GLY	214.61	1292.55 ± 967.92
ALA	172.11	3722.25 ± 394.67
VAL	113.07	1742.05 ± 91.80
ASP	234.97	3903.36 ± 213.42
HSE	94.60	1721.95 ± 167.81
THR	25.37	862.88 ± 41.37
LEU	14.50	157.39 ± 9.10
PRO	322.62	9532.64 ± 497.28

#### 2.4.4. Sensitivity Analysis

Finally, we addressed the question of the extent to which each input variable, including its inherent uncertainty, contributes to the variance of each intracellular metabolite concentration. For this purpose, a sensitivity analysis was performed with respect to the variances in the input variables, where each single variance was independently reduced to about 50% of the experimentally estimated value. 

**Figure 5:**
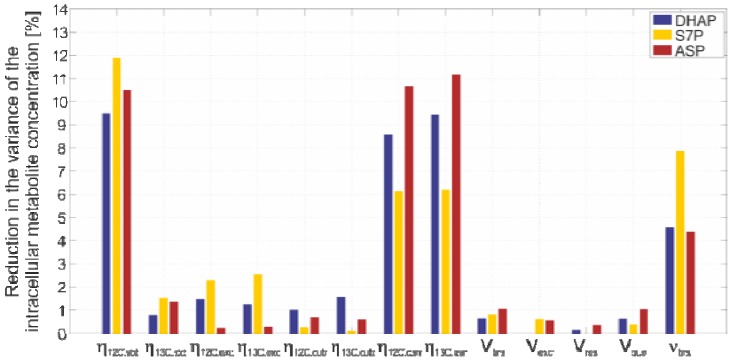
Sensitivity of the variance of each input variable to the variance of the intracellular metabolite concentration. The decrease of every single variance value to about 50% of the experimentally estimated variance leads to a reduction of the final variance represented in the bars.

The simulation results are shown in Figure 5, with the example of three metabolites from different parts of the central metabolism. It can be seen that the input variables with the greatest influence are the ^12^C and ^13^C peak area measurements, resulting from the standard and quenching supernatant samples, followed by the measurement of the biovolume.

Consequently, on the basis of this protocol, the precision of intracellular metabolite data can be further increased by increasing the precision of the LC-MS/MS, as well as the biovolume measurements. Clearly, the precision of both measurements can be improved by increasing the sensitivity of the underlying detector devices at the back end of the measurement method, *i.e.,* mass spectrometer and Coulter counter, respectively. In case of the LC-MS/MS analytics, the matrix effects, although their systematic effect is corrected by applying IDMS, can still have a great impact on the measurement precision. Here, it is conceivable that a further optimization of the liquid chromatography part with respect to a better separation of all analytes will further decrease these matrix interferences. 

## 3. Experimental Section

### 3.1. Strain and Media

In this study, the L-lysine producer strain *C. glutamicum* DM1800 [24] was cultivated in defined glucose medium CGXII [25] containing per liter of distilled water: 20 g (NH_4_)_2_SO_4_, 1g K_2_HPO_4_, 1 g KH_2_PO_4_, 5 g urea, 10 g D-glucose, 13.25 mg CaCl_2_*2H_2_O, 0.25 g MgSO_4_*7H_2_O, 1 mg FeSO_4_*7H_2_O, 1 mg MnSO_4_*H_2_O, 0.02 mg NiCl_2_*6H_2_O, 0.313 mg CuSO_4_*5H_2_O and 1 mg ZnSO_4_*7H_2_O. The medium was adjusted to a pH of 7.0 with sodium hydroxide. The medium additionally contained 3 mL of 10% (v v^−1^) AF 204 (Sigma) and 1 mL of a 0.2 g L^−1^ biotin stock solution, which were added after sterilization. Cryocultures were stored at −80°C in CGXII medium containing 20% (v v^−1^) glycerol.

### 3.2. Cultivation Conditions

Batch cultivation was performed in a 1.5 l bioreactor (DASGIP AG, Jülich, Germany) with a working volume of 1 l. Cells were directly inoculated with 2 mL of cryoculture without performing any pre-culture. The air flow (1 vvm) and temperature (30 °C) were kept constant. The pH was maintained at 7.0 by adding 4 M NaOH and 4 M HCl. Aerobic process conditions (dissolved oxygen > 30%) were ensured via stirrer speed control (200–1200 rpm). During cultivation, the optical density, glucose concentration, cell dry weight, cell count and cell size were measured offline. Dissolved oxygen (Visiferm DO 225, Hamilton Bonaduz AG, Bonaduz, Switzerland), pH (405-DPAS-SC-K80/225, Mettler Toledo GmbH, Gießen, Germany) and exhaust gas concentrations of carbon dioxide and oxygen (GA4, DASGIP AG, Jülich, Germany) were measured online.

### 3.3. Sampling and Sample Processing

For culture broth analysis, a cell suspension volume of 2 mL was drawn into a 5 mL plastic syringe and then dropped to withdraw the dead volume of the sample port. Then 3 mL was drawn into a fresh 5 mL plastic syringe. 1 mL was centrifuged (60 s, 13.000 g, Biofuge pico, Thermo Fisher Scientific, Waltham, USA), and the supernatant was squeezed through a sterile filter (polyvinylidene fluoride, 0.2 µm pore size, Dia-Nielsen, Düren, Germany). From this filtrate, 250 µL was transferred to 750 µL of −20 °C 60% (v v^−1^) methanol for LC-MS/MS analysis and 50 µL was used for glucose analysis.

For the analysis of intracellular metabolites, 6 mL of culture was drawn into a 10 mL plastic syringe and placed in a beaker. Then, 5 mL of the sample was transferred to a reaction tube containing 15 mL of −60 °C 60% methanol. The sample and the quenching solution were mixed by vigorous shaking. Afterwards, the quenched cells and the quenching supernatant were separated via centrifugation (10 min, 9,500 g, −20 °C, Avanti 30, Beckman Coulter, Krefeld, Germany), the supernatant was decanted, filtrated (0.22 µm cellulose acetate, Dia-Nielsen) and stored at −20 °C until analysis. The pellet was resolved in −20 °C methanol and 4 °C TE buffer (10 mM Tris, 1 mM EDTA, pH 7.0). The volume of the extraction media (TE buffer and methanol) was dependent on the biovolume in the pellet, since the extraction of metabolites from 1 µL of biovolume requires 10 µL of methanol and 10 µL of TE buffer. After the addition of an equal volume of −20 °C chloroform, the sample was incubated for 2 h at −20 °C in a Labquake shaker (Reax 2, Heidolph, Schwabach, Germany). All samples were stored at −20 °C until analysis.

For the measurement of optical density, the cell dry weight, cell count and biovolume full samples were taken by drawing the required volume into a plastic syringe after withdrawal of the dead volume.

### 3.4. Offline Analysis

Optical density (OD_600_) was measured at 600 nm (PharmaSpec UV 1700, Shimadzu, Duisburg, Germany) against 0.9% (w v^−1^) NaCl. Glucose was measured using an enzymatic analysis system (EBIO compact, Eppendorf AG Hamburg, Germany). Cell count and cell size were measured using a Coulter counter equipped with a 45 µm capillary (CASY^®^ 1 Modell TT, Roche Diagnostics, Mannheim, Germany). Cell dry weight was measured gravimetrically by placing 2 mL of a full sample in a reaction tube of known dry weight. The cells were separated by centrifugation (2 min, 13.000 g, Biofuge pico, Haereus), dried for 48 h at 80 °C and chilled to room temperature in a desiccator (Neubert-Glas, Geschwenda, Germany).

### 3.5. Metabolome Analysis

Culture supernatants and cell extracts were measured by HPLC (X-LC 3000 Series, Jasco, Tokyo, Japan) coupled to a mass spectrometer (API 4000, ABSciex, Framingham, USA) equipped with a TurboIon spray source. For the analysis of intermediates of the central metabolism, a C18 column (Synergi Hydro, Phenomenex, Aschaffenburg, Germany) was used with eluent A (10 mM tributylamine aqueous solution adjusted pH to 4.95 with 15 mM acetic acid) and eluent B (methanol) at a temperature of 60 °C. The elution gradient was as follows: 2 min (100% A), 5 min (80% A), 8 min (80% A), 10 min (65%), 14 min (0% A), 15 min (0% A), 15.5 min (100% A) and 17 min (100% A). Amino acids were analyzed by applying an ion exchange column (Luna SCX, Phenomenex) with eluent A (5% acetic acid) and eluent B (15 mM ammonium acetate aqueous solution adjusted pH to 6.0 with 100% acetic acid) at a temperature of 40°C. The elution gradient was as follows: 10 min (85% A), 17 min (0% A), 25 min (0% A) and 29 min (85%). In both cases, the flow rate was 0.45 mL min^−1^ and the injection volume 10 μl. For details regarding MS operation see [26,27]. Both methods were used with a ^13^C-labeled internal standard applying the IDMS method [20]. The internal standard was produced by performing a batch cultivation of *E. coli* with uniformly ^13^C-labeled glucose as the sole carbon source.

### 3.6. Estimation of Systematic and Random Errors

The errors of all volumetric process quantities (sample, quenching and extraction volume) were determined gravimetrically by aliquoting the pre-defined volumes 20 times in reaction tubes of known weight. The aliquotation was done using the same instruments (pipet and syringe) used for the metabolome analysis. From the weight differences, mean values and variances were estimated to correct for biases and permit error propagation analysis. 

To estimate the precision of the LC-MS/MS measurement as a function of the matrix complexity, all relevant sample types (standard, extract, quenching supernatant and culture supernatant sample) were measured in five replicates. Here, glucose-6-phosphate, fructose-6-phosphate, phosphoenolpyruvate, pyruvate, acetyl-CoA, citric acid, cis-aconitate, 2-oxoglutaric acid, succinic acid and N-acetyl serine were chosen as examples of metabolites. The variances of the integrated peak areas were determined for each ^12^C mass isotope of the analyte, as well as the corresponding ^13^C mass isotope of the internal standard. The extract, quenching and culture supernatant samples were taken from a cultivation of *C. glutamicum* DM1800.

The variances of cell count, biovolume and cell dry weight measurements were determined by 20-fold replicate measurement of cell samples taken from a cultivation of *C. glutamicum* DM1800.

## 4. Conclusions

For all measurements, in particular quantitative ones, a quantitative indicator of the quality of the result is required to assess its reliability. This is not only vital for subsequent interpretation and modeling purposes, but also to allow a reliable comparison of the results. In our work, we detailed the metabolome data processing workflow according to a specific protocol. 

A model is formulated that enables the quantitative analysis of metabolome data processing. The accuracy of the concentration measurements is studied with respect to uncertainties in the input variables. The model allows us to identify the most critical process steps and is therefore suitable for designing a more robust metabolome data processing pipeline. 

Moreover, our model-based approach is general in the sense that it can be readily transferred to alternative processing workflows to evaluate and express the inherent uncertainties of the basic building blocks as well as the overall uncertainty.
